# Diagnostic Value of Opportunistic CT-Based Bone Density Assessment in Patients with and Without Sacral Insufficiency Fractures

**DOI:** 10.3390/diagnostics15222926

**Published:** 2025-11-19

**Authors:** Julian Ramin Andresen, Guido Schröder, Thomas Haider, Hans-Christof Schober, Reimer Andresen

**Affiliations:** 1 Division of Orthopaedics, Department of Orthopaedics and Trauma Surgery, Medical University of Vienna, Währinger Gürtel 18-20, 1090 Vienna, Austria; 2 Clinic for Orthopaedics and Trauma Surgery, Sana Hospital Bad Doberan, Academic Teaching Hospital of the University of Rostock, Am Waldrand 1, 18209 Hohenfelde, Germany; 3 Division of Trauma Surgery, Department of Orthopaedics and Trauma Surgery, Medical University of Vienna, Währinger Gürtel 18-20, 1090 Vienna, Austria; 4 Practice for Orthopaedics and Osteology, OrthoCoast, Hufelandstraße 1, 17438 Wolgast, Germany; 5 Institute for Diagnostic and Interventional Radiology/Neuroradiology, Westküstenklinikum Heide, Academic Teaching Hospital of the Universities of Kiel, Lübeck und Hamburg, Esmarchstraße 50, 25746 Heide, Germany

**Keywords:** cancellous bone density, Hounsfield units, osteoporosis, risk factors, sacral insufficiency fracture, vitamin D deficiency

## Abstract

**Background/Objectives:** This retrospective observational cohort examined whether opportunistic CT-derived Hounsfield units (HU) of the lumbar spine and proximal femur together with serum 25-OH-vitamin D can predict sacral insufficiency fractures (SIF) and osteoporosis. No interventional procedures were performed. **Methods:** Consecutive suspected SIF cases over 3 years (*n* = 253) were assigned to SIF (*n* = 98) or controls without SIF or spine/hip fractures (*n* = 155). HU were measured using ellipsoidal ROIs at L1–L3 and an irregular area ROI across the entire proximal femoral cancellous bone; vitamin D was quantified; ROC analyses assessed discrimination. HU cut-points were referenced via HU-to-QCT/CTXA conversions. **Results:** SIF patients had markedly lower HU than controls (lumbar 44.84 vs. 105.66 HU; femoral 47.0 vs. 148.0 HU). Diagnostic performance was excellent (AUC 0.98 for SIF discrimination using lumbar HU; AUC 0.98 for osteoporosis prediction using femoral HU). Vitamin D deficiency (<20 ng/mL) was highly prevalent (92.9%) with lower means in SIF (3.72 vs. 8.24 ng/mL). Within SIF, patients with hip fracture had femoral HU ≈ 14.2 vs. 70.6 without hip fracture; effect sizes were very large. **Conclusions:** Opportunistic HU assessment from routine CT provides a rapid, reproducible surrogate of bone density that distinguishes SIF with near-perfect accuracy and identifies osteoporosis. HU thresholds around ~96–98 are consistent with osteoporotic ranges and can be implemented to trigger metabolic evaluation and early osteoanabolic therapy where appropriate.

## 1. Introduction

Sacral insufficiency fractures (SIF), also known as fragility fractures of the sacrum (FFS), occur in patients with reduced bone quality, for example, as a result of rheumatoid arthritis, renal osteodystrophy, secondary hyperparathyroidism due to vitamin D deficiency with consecutive osteomalacia, or ankylosing spondylitis. In addition, SIFs are increasingly observed after corticosteroid therapy or pelvic radiation. Older postmenopausal women with osteoporosis are at the highest risk [1,2,3,4,5,6,7,8,9,10]. For this risk group, an incidence of 2–5% is assumed [11,12]; a significantly higher rate can be assumed for people over 80 years of age, but no valid data is available on this. Against the backdrop of demographic developments, according to which the proportion of people over 80 will almost double by 2040 [13], a significant increase in SIF cases is to be expected [7,14,15,16], with an increase in incidence of 37.5% already recorded in the Netherlands between 1986 and 2011 [16].

Since it was first described by Lourie in 1982 [9] in three patients with osteoporosis, this type of fracture is increasingly being diagnosed due to increased clinical awareness [14,15,16] and improved imaging techniques [17,18,19]. The previously limited sensitivity of conventional imaging [11] could be further improved in the future through the use of artificial intelligence [20].

Fractures of the anterior pelvic ring or the ala ossis ilii often occur concomitantly. Conversely, in more than half of cases where a fracture of the anterior pelvic ring is detected, a SIF is already present [19,21,22,23,24,25]. SIFs usually occur spontaneously or as a result of low-energy minor trauma [26,27] and are considered indicator fractures of clinically manifest osteoporosis [22]. After lumbosacral fusion, they represent an increasing complication due to the altered biomechanics of the spine [28].

Clinically, pronounced, immobilizing pain in the lower back, buttocks, and groin, as well as local pressure pain above the fracture zone, predominate [24,25,26,27,29,30]. Inability to stand or walk with progressive immobilization occurs particularly in bilateral fractures. Neurological deficits are rare and typically manifest as isolated S1 syndrome or cauda equina syndrome limited to the sacral nerve roots [31].

Persistent immobilization promotes complications such as deep vein thrombosis with pulmonary artery embolism, pressure ulcers, infections, or an increase in osteosarcopenia, which is associated with increased morbidity [32] and a mortality rate of up to 23.5% within twelve months [33]. Treatment depends on clinical symptoms and fracture morphology, with a focus on stability/instability, and is conservative, involving sacroplasty or osteosynthesis [34,35]. The classification of fragility fractures of the pelvis (FFP) according to Rommens and Hofmann has become established for fracture classification [21].

Prior HU-based studies have shown that lumbar spine Hounsfield units (HU) from opportunistic CT correlate with bone mineral status and vertebral fragility, while proximal femoral HU discriminates hip fracture risk. However, most investigations evaluated HU in isolation and rarely incorporated biochemical markers of bone health. We advance this discourse by integrating opportunistic HU analysis from the lumbar spine and proximal femur with serum 25-OH-vitamin D to provide a more clinically actionable signal for SIF. Can routinely acquired CT data provide reliable bone density surrogates that correlate with SIF and vitamin D deficiency? Accordingly, we conducted a retrospective cohort study to test whether opportunistic HU analysis of the lumbar spine and proximal femur, together with serum 25-OH-vitamin D, discriminate SIF and identify osteoporosis with clinically actionable thresholds.

## 2. Materials and Methods

An ethics vote from the medical faculty of the responsible university with the number: D 471/24, dated 28 March 2024, is available for retrospective data collection. Patient consent was waived due to it not being an experimental study. It is a non-interventional, retrospective clinical analysis of pseudonymized routine patient data and existing CT images. No additional imaging, treatment, randomization, or patient contact was performed.

Over the last three years, 253 consecutively recorded patients with clinical suspicion of SIF were retrospectively analyzed.

Taking into account the imaging performed–conventional X-ray (pelvis a.p.); computed tomography (CT) with an axial slice thickness of 2 mm through the pelvis and coronal reformed slices of 2 mm angulated to the sacrum, each documented in the bone and soft tissue window; and magnetic resonance imaging (MRI) of the pelvis with axial and sagittal T1- and T2-weighted 4 mm slice images and a STIR sequence (slice thickness 2.8 mm) angled coronally to the sacrum—the patients were divided into two groups: group 1: 98 patients with SIF and additional fractures in the spine and/or hip; control-group 2: 155 patients without SIF and without fractures in the spine or hip.

The mean age was 73.85 (61–92) years overall, 72.28 (61–85) years for men, and 74.11 (61–92) years for women. The mean BMI was 25.15 (17.6–37.6) kg/m^2^ overall, 27.62 (20–37.6) kg/m^2^ for men (–37.6), and 24.74 (17.6–34.4) kg/m^2^ for women. The age and BMI of all patients and of groups 1 and 2 are summarized in Table 1.

Patients with a condition following high-energy trauma, known malignancy, and condition following spondylodesis as well as bilateral hip TEP were excluded from the study.

In addition to age, gender, and BMI, vitamin D levels, pain according to VAS upon admission to SIF, other possible risk factors, and other fractures recorded in the medical history were also recorded.

### 2.1. Detection of Fractures

If an additional hip fracture was suspected, a conventional X-ray was taken in two planes. The hip fractures recorded include femoral neck and pertrochanteric fractures.

To detect fractures of the axial skeleton, X-rays of the thoracic and lumbar spine were taken in two planes. Spinal fractures were classified according to their location and quantified according to the degree of deformation using Genant et al. classification [36].

Sacral fractures were classified according to the Fragility Fractures of the Pelvis (FFP) classification by Rommens and Hofmann, taking into account CT and MRI [21]. Four fracture types with several subgroups are assigned to the pelvic ring.

FFP Type I: The fractures are stable. Type I describes unilateral (a) and bilateral (b) anterior pelvic ring fractures.

FFP Type II: The fractures are moderately stable. Type II describes the non-displaced sacral fracture (a), fracture in the lateral mass of the sacrum with anterior pelvic ring fracture (b), and complete vertical non-displaced sacral fracture with anterior pelvic ring fracture (c).

FFP Type III: The fractures are unstable. Type III describes a displaced unilateral ilium fracture (a), a displaced iliosacral injury (b), and a displaced unilateral sacrum fracture (c).

FFP Type IV: The fractures are highly unstable. Type IV describes a displaced bilateral ilium fracture (a), a displaced bilateral sacrum fracture with transverse fracture (b), and combined dorsal fractures with anterior pelvic ring involvement (c).

### 2.2. Determination of Bone Density

**Figure 1:**
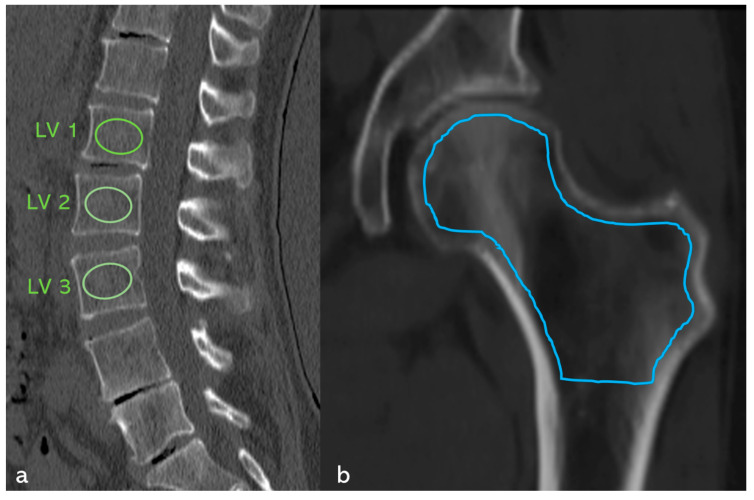
Measurement of cancellous density in HU. (**a**) Sagittal reformatted CT slice of the lumbar spine with positioning of the ellipsoidal ROI (green) in the mid-vertebral layer of LV1, LV2, and LV3. (**b**) Coronary reformatted CT slice of the left hip with positioning of the irregular ROI (blue) in the proximal femur.

Cancellous bone density was determined using Hounsfield units (HU) in CT (GE Revolution^TM^ EVO/64 line CT–GE Health Care GmbH, Düsseldorf, Germany) in all patients. In the spinal region, the density in HU was determined by manually positioning an ellipsoidal ROI in the mid-vertebral spongy space in the sagittal reformatted CT slice at the level of LV1 to LV3, with a layer thickness of 2 mm and a window setting of C = 400/W = 1600 [37] (Figure 1a). If a fracture was present in this segment, the adjacent vertebral bodies were used instead.

In the hip region, the cancellous density in HU was determined manually in the largest coronary CT slice using an irregular area ROI across the entire proximal femur region (Figure 1b). The distance to the cortex was set at approximately 1 mm. All examinations were performed with a CT tube voltage of 120 kV; the HU were measured with a layer thickness of 2 mm and a window setting of C = 400/W = 1600. This concept had already been successfully applied and modified in a previous study [38].

### 2.3. Treatment of the Patient

Taking into account the general situation, the immobilizing pain, and the fracture classification, patients with a SIF were referred for conservative, interventional, or osteosynthetic treatment in accordance with a therapeutic flowchart [34]. Patients with a hip fracture were or had already been treated in accordance with guidelines.

To minimize the existing risk of fracture and inhibit further bone destruction, anti-osteoporotic therapy, preferably osteoanabolic, was recommended in accordance with the DVO guidelines [39].

### 2.4. Statistical Analysis

The data were analyzed using SPSS Statistics, version 23.0 (IBM Corp., Armonk, NY, USA).

Quantitative characteristics are presented as mean ± standard deviation (M ± SD; additionally, *n*) for parametric distribution and as median (Q1–Q3) for nonparametric distribution.

Group comparisons (without vs. with SIF) were performed using the Welch t-test; additionally, exploratory analyses were performed using the Mann–Whitney U test. The test selection was based on the Shapiro–Wilk test for normal distribution. All *p*-values are two-sided; *p* < 0.05 is considered significant, *p* < 0.005 highly significant, and *p* < 0.0005 very highly significant.

For small-volume groups, the effect size was reported according to Hedges’ g; 0.20 = small effect, 0.50 = medium effect, 0.80 = large effect.

ROC analysis was used to determine the predictive power for fractures and osteoporosis based on the HU values of the lumbar spine (ellipsoidal ROI LV 1–3, Figure 1a) and the hip (irregular area ROI of the entire femoral region, Figure 1b), and optimal cut-points were determined using the Youden index, with secondary thresholds chosen to reflect a sensitivity/specificity balance.

## 3. Results

### 3.1. Patients

See Table 1 for age distribution and BMI.

Of a total of 253 patients, 98 patients had SIF (group 1), comprising 14 men (14.3%) and 84 women (85.7%).

The overall average age of the group was 73.85 years. The age of patients in group 1 (78.95 ± 8.33) was 8.33 years higher than that of patients in group 2 (70.62 ± 6.21) (95% CI −10.26 to −6.40) with a *p* < 0.001. 70.62 ± 6.21, by 8.33 years (95% CI −10.26 to −6.40) with a *p* ≤ 0.001, which is significantly higher; the effect size is 1.84.

For men: group 1: *n* = 14, 74.14 ± 8.97 compared to group 2: *n* = 22, 71.09 ± 5.70; difference = 3.05 years (95% CI −8.66 to 2.56), *p* = 0.27, effect size 0.42, the age is not significantly different.

**Figure 2:**
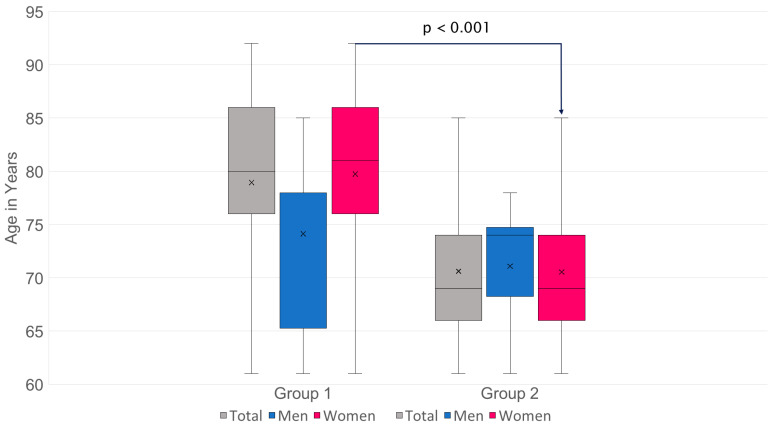
There is an age difference in favor of a higher age in group 1, which is predominantly attributable to women.

For women: group 1: *n* = 84, 79.75 ± 8.00 compared to group 2: *n* = 133, 70.54 ± 6.31; difference = 9.21 years (95% CI −11.25 to −7.17), *p* < 0.001, effect size 1.31, the age is clearly significantly different (Figure 2).

Overall, the collective has an average BMI of 25.15 kg/m^2^. The BMI of group 1: *n* = 98, 24.92 ± 4.41 kg/m^2^ is compared with the patients in group 2: *n* = 155, 25.30 ± 3.96 kg/m^2^; difference = 0.38 kg/m^2^ (95% CI −0.70 to 1.46), *p* = 0.49, not significantly different, the effect size is 0.12.

For men: group 1: *n* = 14, 27.97 ± 5.30; group 2: *n* = 22, 27.40 ± 5.52; difference = 0.57 (95% CI −4.09 to 2.95), *p* = 0.74, effect size 0.11, the BMI is not significantly different.

**Figure 3:**
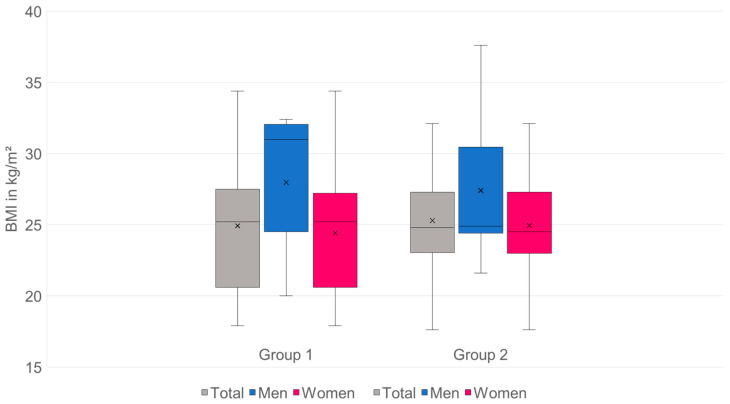
There is no significant difference in BMI between group 1 and group 2, either overall or by gender.

For women: group 1: *n* = 84, 24.41 ± 4.18; group 2: *n* = 133, 24.95 ± 3.56; difference = 0.54 (95% CI −0.55 to 1.62), *p* = 0.33, effect size 0.14, the BMI is not significantly different (Figure 3).

### 3.2. Fracture Morphology and Fracture Distribution

#### 3.2.1. Pelvis: Classification of SIFs According to FFP (Figure 4)

FFP type II accounted for 61.3% of fractures, while FFP type IIIc, type IVb, and type IVc together accounted for 38.7%.

**Figure 4:**
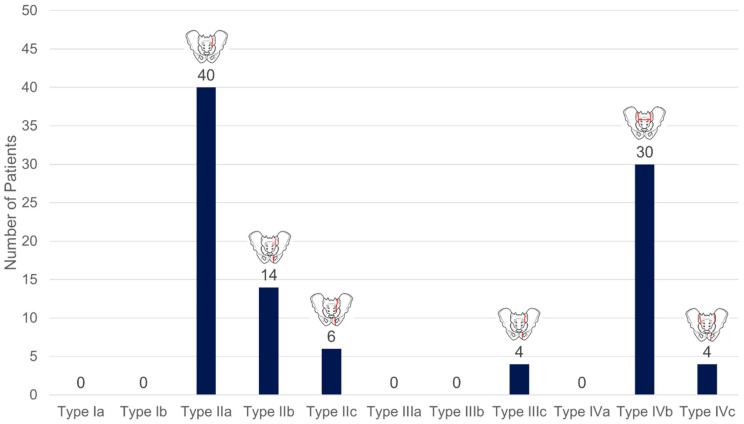
Number of patients with corresponding FFP types according to Rommens & Hofmann [21].

#### 3.2.2. Hip Fractures (Figure 5)

Of the 16 femoral neck fractures, 36.7% were in women with an average age of <75 years. Of the 14 pertrochanteric fractures, 64.3% were in women with an average age of >75 years.

**Figure 5:**
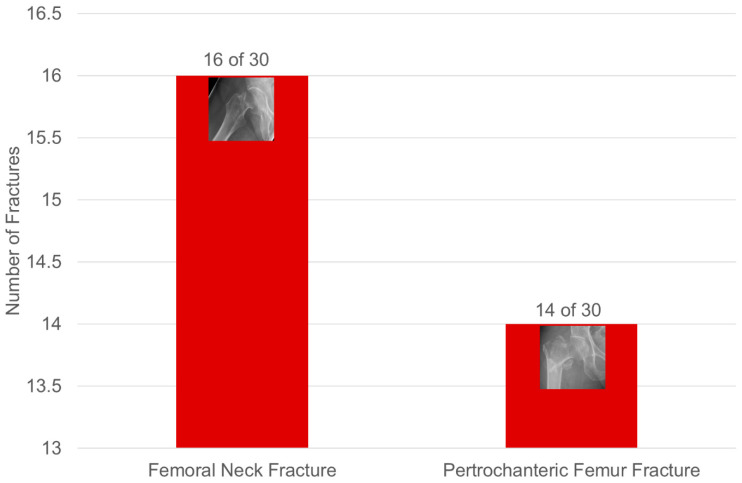
Distribution of hip fractures in patients in group 1.

#### 3.2.3. Fractures of the Axial Skeleton (Figure 6, Table 2)

There is a concentration of fractures at the vertex of the thoracic kyphosis, thoracolumbar and sacral.

**Figure 6:**
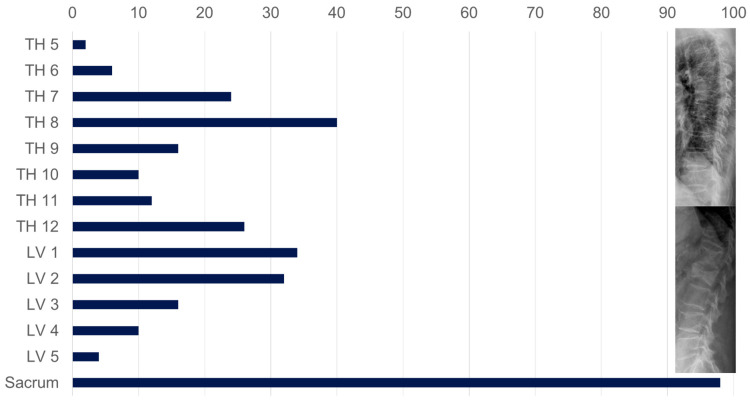
Distribution of vertebral fractures in patients in group 1.

**Table 2:** Classification of vertebral deformities and SIF for patients in group 1.

	Patients (*n* = 98)	Men (*n* = 14)	Women (*n* = 84)
Age (in years)	Ø 78.95	Ø 74.14	Ø 79.75
	(min.: 61; max.: 92)	(min.: 61; max.: 85)	(min.: 61; max.: 92)
BMI (kg/m^2^)	Ø 24.92	Ø 27.97	Ø 24.41
	(min.: 17.9; max.: 34.4)	(min.: 20; max.: 32.4)	(min.: 17.9; max.: 34.4)
Patients with at least one fracture (*n*)	98	14	84
Average OVF accumulation per patient	2.37	2.71	2.31
Affected vertebral bodies (Figure 6) (*n*) with at least one grade 1 vertebral body deformity according to Genant et al. [36]	232	38	194
SIF	98 (100%)	14 (100%)	84 (100%)
Unilateral	14 (14.29%)	0	14 (16.67%)
on both sides	84 (85.71%)	14 (100%)	70 (83.33%)

### 3.3. Comparison of Cancellous Bone Density in HU of the Lumbar Spine and Proximal Femur in Group 1 and Group 2

For the lumbar spine:

- *n*: G1 = 98·G2 = 155.
- Mean values (SD): G1 44.84 (18.28) HU·G2 105.66 (27.67) HU.
- Mean difference (G2−G1): +60.82 HU·95% CI: [+55.13; +66.51].

Significance:

- Welch’s *t*-test: t = −21.05, df ≈ 250.5, *p* = 2.50 × 10^−57^.
- Mann–Whitney U: U = 417, *p* = 1.04 × 10^−37^.

**Figure 7:**
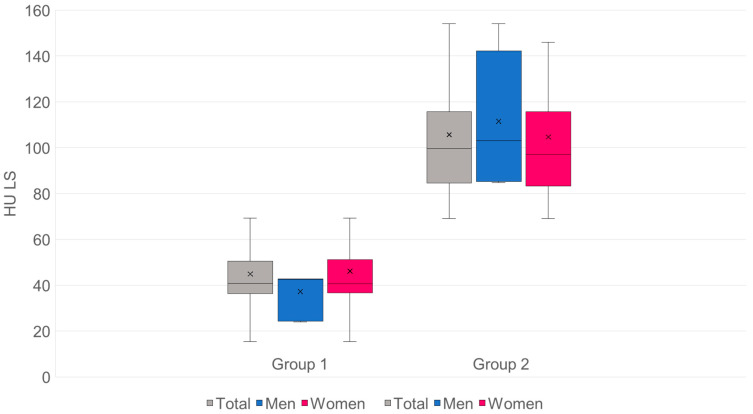
The HU values in the lumbar spine of group 1 are highly significant (*p* < 0.0005) lower than group 2.

Effect size:

- Hedges’ g = 2.48.
- Cliff’s δ = 0.96 → approx. 96% of pair comparisons: value from G2 > G1 (Figure 7).

The mean HU value for group 1 is 44.84, which corresponds to 37.16 mg/cm^3^.
The mean HU value for group 2 is 105.66, which corresponds to 86.42 mg/cm^3^.
The threshold for osteoporosis of 80 mg/cm^3^ is 97.73 HU.

For the proximal femur:

- *n*: G1 = 98·G2 = 155.
- Mean values (SD): G1 47 (34.5) HU·G2 148.0 (49.3) HU.
- Mean difference (G2−G1): +101.0 HU·95% CI: [+90.6; +111.4].

Significance:

- Welch’s *t*-test: t = −19.13, df = 248.4, *p* = 9.09 × 10^−51^.
- Mann–Whitney U: U = 520, *p* = 9.95 × 10^−36^.

**Figure 8:**
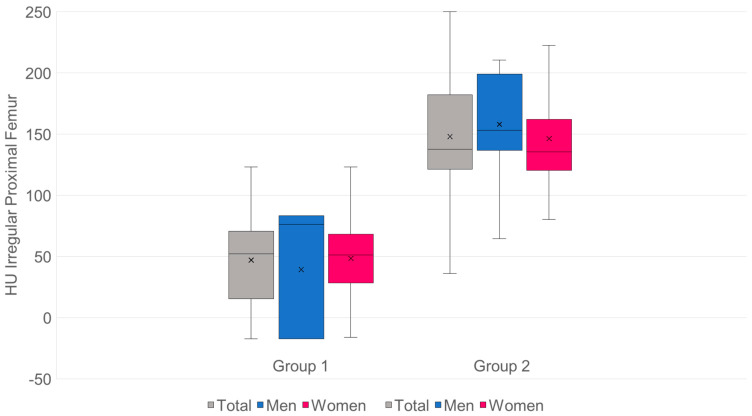
The HU values in the proximal femur of group 1 are highly significant (*p* < 0.0005) lower than group 2.

Effect size:

- Hedges’ g = 2.28.
- Cliff’s δ = 0.93 → approx. 93% of pair comparisons: value from G2 > G1 (Figure 8).

The mean HU value for group 1 is 47, which corresponds to a T-value of −3.58.
The mean HU value for group 2 is 148, which corresponds to a T-value of −1.35.
The threshold for osteoporosis with a T-value of −2.5 is 95.93 HU.

#### Spongy Bone Density of Patients with and without Hip Fractures in Group 1 (Figure 9)

Descriptive: With proximal femur fracture: mean 14.2 HU (SD 21.4), median 12.1 (IQR 25.2), min −17.3, max 47.9; without proximal femoral fracture: mean 70.6 HU (SD 19.5), median 68.1 (IQR 28.4), min 38.6, max 123.1.

**Figure 9:**
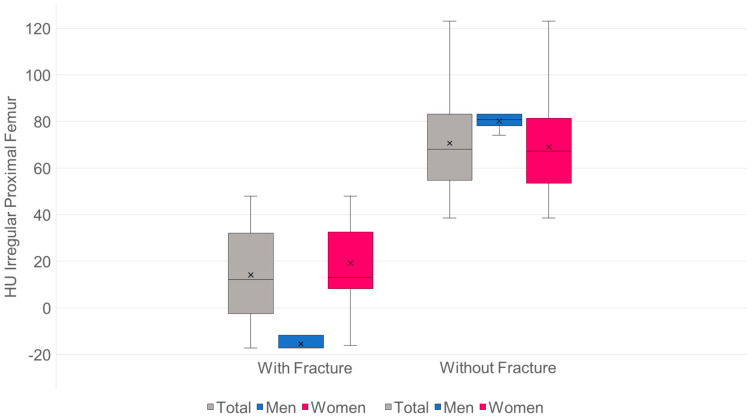
Hip fractures.

Distribution & tests: Normality not given (Shapiro–Wilk: with *p* < 0.001; without *p* = 0.02). Mann–Whitney U: U = 2304.5; *p* < 1 × 10^−12^.

Effect size (rank-based): Cliff’s delta = 0.972 (extremely large; almost all values in “without” > “with”). Robustness check (parametric, Welch-t): t = 13.37; *p* < 1 × 10^−12^; Hedges g = 2.76. Mean difference: +56.5 HU in favor of “without”; 95% CI (bootstrap): [48.4; 64.6].

Interpretation: Patients without proximal femoral fractures have an average of approximately 56 HU more than those with fractures—statistically and practically very significant. The distributions overlap only minimally (max “with” 47.9 vs. min “without” 38.6).

In the subgroup analysis within the patient cohort with sacral fractures, patients with proximal femoral fractures had significantly (*p* < 0.001) lower HU values than those without proximal femoral fractures. The mean HU value of 14.2 for patients with a proximal femur fracture corresponds to a T-value of −4.3, while the mean HU value of 70.6 for patients without a proximal femur fracture corresponds to a T-value of −3.1.

**Figure 10:**
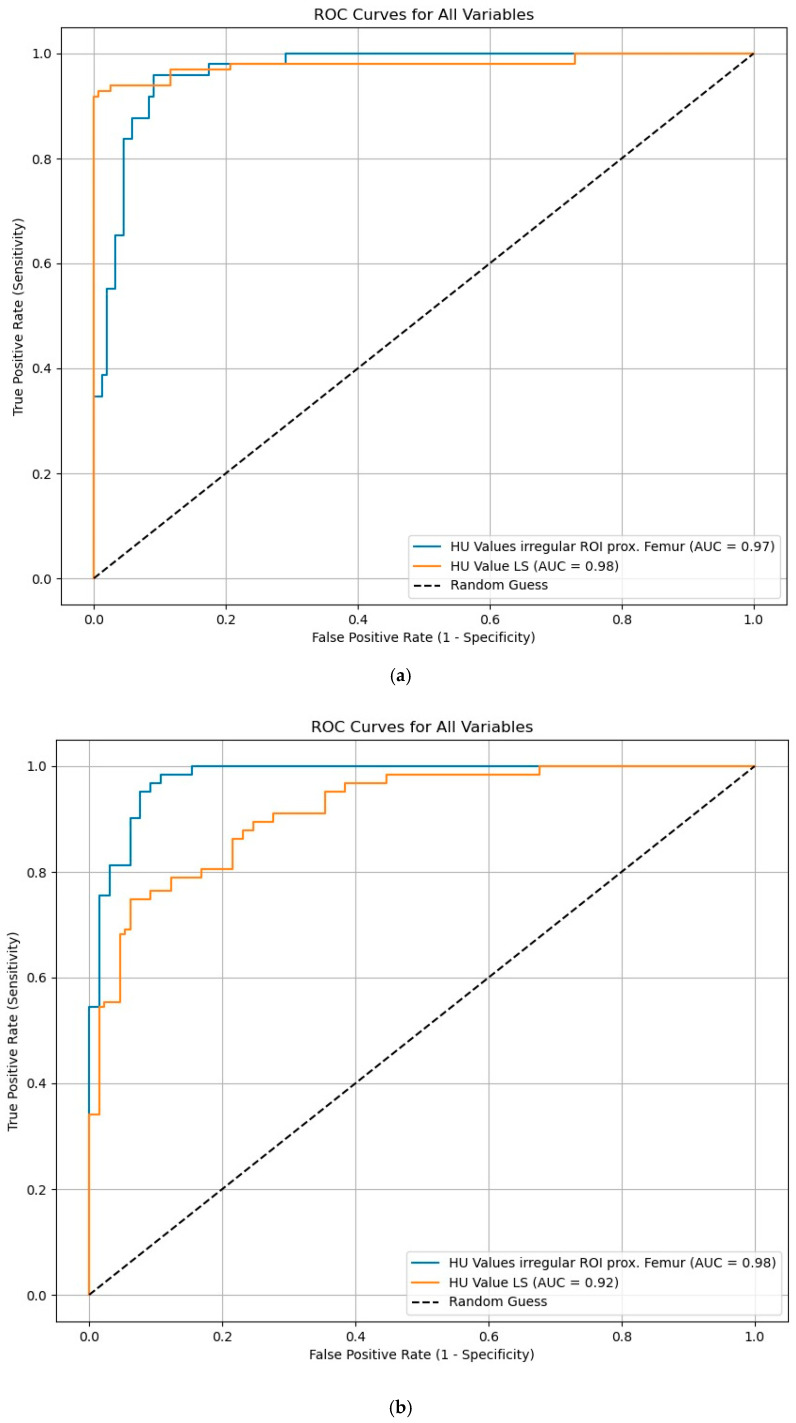
(**a**) ROC curve analysis shows that HU values of the lumbar spine with an AUC = 0.98 have a slightly higher fracture predictive power for SIF than the HU values from the irregular area ROI of the proximal femur with an AUC = 0.97. This is found at an effect size of 2.83. However, each region is suitable for reliably assigning a risk for SIF in practice. (**b**) ROC curve analysis shows that HU values of the irregular area ROI of the proximal femur with an AUC = 0.98 have a higher osteoporosis prediction power than the HU values of the ellipsoidal ROIs in the lumbar spine with an AUC = 0.92. This is found at an effect size of 2.81. However, each region is suitable for reliably assigning a possible osteoporosis.

The predictive power for fractures is shown in Figure 10a and the predictive power for osteoporosis in Figure 10b.

### 3.4. Conversion of HU Values into Equivalent Quantitative QCT Values in mg/cm^3^ for the Lumbar Spine and Equivalent Quantitative CTXA Values in mg/cm^2^ (Table 3) and Comparison to a Previous Study on the Hip (Table 4)

To convert the HU values into equivalent quantitative QCT values in mg/cm^3^, we used the equation from the correlation coefficients of Andresen et al., 2025 [37]. The osteoporosis threshold value of 80 mg/cm^3^ [39] is 97.7 HU in this case.

**Table 3:** Comparison of methodologically comparable studies on osteoporosis determination in the lumbar spine.

Conversion Values from Different Studies			
Studies	Average Age (in Years)	Conversion Formula from HU to QCT Values (mg/cm3)	Absolute Value and Percentage Deviation at 100 HU Compared to Own Results
Buenger et al., 2021 [40]	66.98	QCT value	87.8 mg/cm^3^
	min.: 28; max.: 92	= 17.8 + (0.7 × HU)	9.6% greater than the values from [37]
Schröder et al., 2024 [41]	81.1	QCT value	73.7 mg/cm^3^
	min.: 66; max.: 102	= 13.7 + (0.6 × HU)	8.7% lower than the values from [37]
Andresen et al., 2025 [37]	65.9	QCT value	81.8 mg/cm^3^
	min.: 24; max.: 91	= 0.84 + (0.8127 × HU)	

**Table 4:** Methodologically comparable studies on osteoporosis determination in the hip/proximal femur region.

Conversion Values from a Previous Study			
Study	Average Age (in Years)	Conversion Formula from HU to CTXA Values T-Score Values	At 100 HU, the Following T-Value Is Obtained
Andresen et al., 2025 [38]	65.9 min.: 24; max.: 91	CTXA value = −4.62 + (0.0221 × HU)	−2.41

To convert the HU values into equivalent CTXA T-scores, we used the equation from the correlation coefficients of Andresen et al., 2025 [38] for our data. The osteoporosis threshold value of a T-score of −2.5 [39] is 95.9 HU in this case.

### 3.5. Vitamin D Levels

Vitamin D deficiency with values < 20 ng/mL was found in 235 (92.9%) of all 253 patients. The vitamin D level for all patients was Ø 5.93 (0–26.1) ng/mL, in group 1 it was Ø 3.72 (0–21.2) ng/mL, and in group 2 it was Ø 8.24 (3.2–26.1) ng/mL. The differences between groups 1 and 2 are significant with *p* < 0.05, g = 2.83.

For group 1, when the vitamin D values were assigned to the individual FFP types, there were no significant (*p* > 0.05) differences between types IIa, IIb, IIc, and IIIc, as well as types IVb and IVc.

### 3.6. Pain at the Onset of SIF

All patients with SIF experienced immobilizing pain. Patients with FFP types IIa to IIc had an average of 7 (5–9) pain points, while patients with FFP types IIIc, IVb, and IVc had an average of 9 (8–10) pain points. The differences are significant with *p* < 0.05.

### 3.7. Additional Disease Profile

In 98 (38.7%) of 253 patients, at least one sintering fracture was found in the thoracic and lumbar spine (Figure 6, Table 2). Additional fractures in the anterior pelvic ring were found in a total of 28 (28.6%) of the 98 patients with a SIF. In 30 (30.6%) of 98, an additional hip fracture was found (Figure 5); these fractures are all from patients in group 1.

Further osteoporosis-associated fractures such as distal radius, proximal humerus, rib, and sternum fractures were found in the medical history of 96 (37.9%) of 253 patients, with significantly more distal radius fractures found in group 1 than in group 2.

Mild hypocalcemia was found in 40.2%, secondary hyperparathyroidism in 42.3%, and elevated ostease-levels in 46.8% of all patients. Ostease-levels themselves were significantly (*p* < 0.05) more frequently > 24 µg/L in group 1 than in group 2.

An additional lung disease was found in 23.6%, cardiovascular disease in 45.2%, arterial hypertension in 77.1%, renal insufficiency in 24.8%, type II diabetes mellitus in 25.8%, peripheral arterial occlusive disease in 22.3%, and obesity with a BMI > 30 kg/m^2^ in 13% of all patients. Varying degrees of nicotine consumption were reported by 34.3% of all patients.

### 3.8. Case Study (Figure 11)

78-year-old patient with clinically manifest osteoporosis, TH 12 sintering fracture, and a history of distal radius fracture. In addition, a new fracture was found in the sacrum on both sides. Clinically, immobilizing pain was rated 9 out of 10 on the VAS scale. No anti-osteoporotic drug therapy had been administered up to this point. The vitamin D level was 2.78 ng/mL.

**Figure 11:**
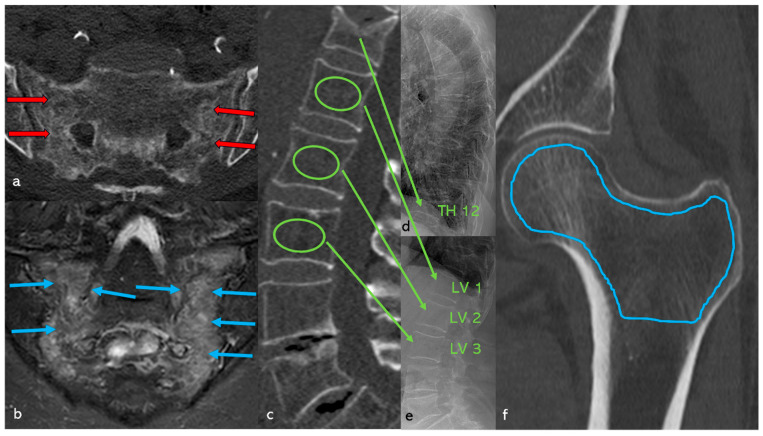
(**a**) Axial CT section of the sacrum. A fracture line with adjacent densification of the cancellous bone (marked with red arrows) is visible on both sides of the lateral mass of the sacrum; no relevant dislocation is found. (**b**) Coronally angulated MRI slice (STIR sequence) of the sacrum. Clear hyperintense edema zones with individual hypointense fracture lines (marked with blue arrows) are visible on both sides. (**c**) Sagittal reformatted CT section of the lumbar spine with positioning of the ellipsoidal ROI (marked with green circles) in the mid-vertebral layer of LV1, LV2, and LV3. The mean value is a cancellous density of 41.4 HU, which corresponds to a correlation coefficient KMG (mg/cm^3^) = (0.8127 × HU) + 0.84 (from Andresen et al. [37]) 35.5 mg/cm^3^, which is clearly within the osteoporotic range [39]. (**d**) Lateral thoracic spine image and (**e**) lateral lumbar spine image showing a TH 12 sintering fracture. (**f**) Coronary reformatted CT section of the left hip with positioning of the irregular ROI (blue line) in the proximal femur. This results in a cancellous density of 34.8 HU, which corresponds to a T-value = (0.0221 × HU) − 4.6169 (from Andresen et al. [38]) −3.85 according to the correlation coefficient. The value is clearly in the osteoporotic range [39].

## 4. Discussion

As in other studies, our patient group with SIF confirmed advanced age [8,15,16], female gender [10,11], osteoporosis [4,33], and severe vitamin D deficiency [4,42] as risk factors. On the other hand, SIF is a strong indicator of the presence of manifest osteoporosis [22]. In our patient group with SIF, silent sintering fractures of the axial skeleton were frequently found (Figure 6, Table 2). These osteoporosis-associated vertebral fractures (OVF) typically occur more frequently in the middle thoracic and thoracolumbar regions [37]. Concurrent hip fractures are associated with further bone loss and occult OVF [43]. As part of osteoporotic bone remodeling, the trabecular structure in the sacrum becomes rarefied and is partially replaced by fatty marrow, which leads to negative HU values in the CT cross-section [42,44,45]. When a sacral fracture occurs, interstitial fluid accumulation, edema, and bleeding into the fracture region cause an increase in density; in CT, an increase of >35 HU can be used to detect occult fractures [46].

As the instability of the SIF increases, there is a significant increase in immobilizing pain [34], which was also evident in our patients with FFP types IIIc, IVb, and IVc.

Taking into account the laboratory parameters and low bone density, there are indications of an osteomalacia component. In a comparable patient cohort, Delsmann et al. [47] found a loss of trabecular microstructure and increased osteoid values with pronounced hypomineralization in biopsies of patients with bilateral sacral insufficiency fractures, which supports this finding.

The aim of our study was to estimate the extent of osteoporosis in patients with and without SIF by determining trabecular bone density (HU) in the lumbar spine and proximal femur in the reformatted CT section. The conversion of HU values into quantitative values (mg/cm^3^) analogous to QCT for the spine [KMG (mg/cm^3^) = (0.8127 × HU) + 0.84] and into T-scores analogous to CTXA for the hip [T-value = −4.62 + (0.0221 × HU)] was performed by adopting correlation coefficients from previously conducted studies [37,38].

In a comparable age group, we were able to show that patients with SIF have a significantly (*p* < 0.001) lower trabecular density (HU) in the lumbar spine and proximal femur than patients without SIF. The HU measurements allow a good assessment of bone status with regard to the presence of manifest osteoporosis [37,38], which can be used as a surrogate parameter for personalized therapy decisions.

### 4.1. The Axial Skeleton

Density measurements on the axial skeleton can be performed in the axial, coronary, or sagittal mid-vertebral plane; the HU values measured in the axial cancellous space tend to be slightly higher than in the coronary and sagittal planes. Kim et al. [48] conclude, based on their cut-off values for osteoporosis (axial 112 HU, coronary 110 HU, sagittal 112 HU), that all three planes can be used equally. In order to compensate for possible density fluctuations in individual vertebral bodies, Scheyerer et al. [49], as we do, also consider it necessary to take measurements on at least three different lumbar vertebral bodies. Overall, these measurements show a high degree of interobserver agreement among experienced examiners [41].

Patients with SIF in our cohort (Figure 7) had significantly lower values (*p* < 0.0005) with mean values of 44.84 HU compared to patients without SIF (105.66 HU).

### 4.2. The Proximal Femur

Density measurements on the proximal femur can be performed in different slice planes and ROI configurations; osteoporosis assessment and hip fracture risk determination are possible, with high agreement with CTXA and DEXA [38,50,51,52,53,54,55,56,57,58]. Compared to DEXA hip, trabecular density determination in HU in the CT cross-sectional image of the femur is free of degenerative overlays and without cortical summation. ROC curve analysis showed that HU values of the entire proximal femur region show a high correlation with the KMG (mg/cm^2^; sensitivity = 0.92; specificity = 0.92) and T-scores (sensitivity = 0.92; specificity = 0.93) at a threshold value of 96 HU in terms of osteoporosis prediction. The determination of HU values across the entire proximal femur region (Figure 1b) showed the highest predictive value (AUC proximal femur = 0.97; AUC caput femoris = 0.93) for osteoporosis prediction compared to the density values of the caput femoris [58], so that only the irregular area ROI was used in the present study. A similar osteoporosis threshold value of 95.93 HU was found (Figure 8). Patients with SIF had significantly lower values (*p* < 0.001) with mean values of 37 HU compared to patients without SIF (148 HU). Patients with SIF and additional hip fractures showed a further significant decrease in density with mean values of 14.2 HU (*p* < 0.001; Figure 9), corresponding to an increasing loss of trabecular structure and an increased risk of hip fracture.

### 4.3. Strengths and Limitations

For patients with SIF, fracture data for the axial skeleton, hip, and periphery were available in parallel. Based on the density values, it was possible to validly classify bone status—a factor with a decisive influence on subsequent therapy. The ROIs on the axial skeleton and proximal femur were collected under identical standardized conditions. A high predictive power was demonstrated both for the estimation of fracture risk (Figure 1a) in SIF and for the presence of osteoporosis (Figure 1b).

A comparison of the HU thresholds with other studies [41,48,50,53,55,56] is only possible to a limited extent, as different ROI sizes, shapes, and positions were used in those studies. Felsenberg et al. [59] reported an average deviation of 2.5% for elliptical, circular, rectangular, and freely defined ROIs in homogeneous, normal-density cancellous bone, as well as a dispersion of 9% in inhomogeneous, osteoporotic vertebral cancellous bone.

Since HU measurement is performed in single-energy mode, it is possible to overestimate the extent of osteoporosis due to a fat error (Table 3). In borderline cases, this can be corrected using QCT in dual-energy mode [54,60].

## 5. Conclusions

Opportunistic HU assessment from routine CT provides a rapid, reproducible surrogate for bone density that distinguishes SIF with excellent accuracy. HU thresholds around 96–98 HU identify osteoporosis and can be readily implemented in radiology workflows to trigger metabolic evaluation and targeted therapy. This can be used in everyday clinical practice without further examinations if CT scans are available. Patients with SIF show HU values in the clearly osteoporotic range. In the case of additional hip fractures, the bone situation deteriorates even further. Furthermore, silent sintering fractures are often found in the axial skeleton, which further confirms the presence of clinically manifest osteoporosis.

Due to the severity of the osteoporosis, we recommend osteoanabolic therapy at least for patient group 1, provided there are no contraindications [61].

Choi et al. [62] show that DEXA measurements tend to provide high density values in cases of degenerative spinal changes, while HU-based determination better reflects the actual situation. CT-based HU values can therefore serve as a complementary method and correct spinal osteoporosis not diagnosed by DEXA [63]. HU thresholds are helpful for assessing the risk of mechanical complications after spinal deformity surgery in adults [64]. After percutaneous balloon kyphoplasty for osteoporotic vertebral fractures (OVF), markedly reduced HU values are predictors of subsequent fractures [65]. The determination of HU values on the spine and proximal femur also influences the assessment of complications; for example, screw loosening can be predicted more accurately with low HU values than with other methods [66,67]. The increase in bone density under osteoanabolic therapy can also be reliably monitored over time using HU measurements [68].

Further research is needed to optimize HU-based thresholds for different patient populations, age groups, genders, and anatomical regions and to enable automated, standardized ROI selection. In addition, data collection and standardized evaluation of HU values from different CT scanners and protocols in heterogeneous populations using AI-based image analysis are promising, including automated segmentation based on machine learning to reduce variability between and within examiners. This would further increase reproducibility and precision and enable and improve the clinical applicability of HU-based osteoporosis diagnostics and fracture risk assessment on a larger scale. Corresponding open-source tools are already available for the hip [69]. A recent review shows that AI-supported evaluations of routine CTs (and X-rays) enable both opportunistic screening and short- to medium-term fracture risk predictions and outperform FRAX without BMD or areal BMD in several cohorts. For clinical implementation, multisite validation and calibration, bias controls, and integration into PACS/HIS with safety mechanisms (fail-safes) and interpretable outputs (e.g., heat maps, survival plots) are required [70].

## Figures and Tables

**Table 1:** Patient description by gender, age, and BMI.

**Characterization of the total sample**			
	Patients (*n* = 253)	Men (*n* = 36)	Women (*n* = 217)
Age	Ø 73.85	Ø 72.28	Ø 74.11
(in years)	(min.: 61; max.: 92)	(min.: 61; max.: 85)	(min.: 61; max.: 92)
BMI	Ø 25.15	Ø 27.62	Ø 24.74
(kg/m^2^)	(min.: 17.6; max.: 37.6)	(min.: 20; max.: 37.6)	(min.: 17.6; max.: 34.4)
**Characterization of group 1 (sacrum fractures)**			
	Patients (*n* = 98)	Men (*n* = 14)	Women (*n* = 84)
Age	Ø 78.95	Ø 74.14	Ø 79.75
(in years)	(min.: 61; max.: 92)	(min.: 61; max.: 85)	(min.: 61; max.: 92)
BMI	Ø 24.92	Ø 27.97	Ø 24.41
(kg/m^2^)	(min.: 17.9; max.: 34.4)	(min.: 20; max.: 32.4)	(min.: 17.9; max.: 34.4)
**Characterization of group 2 (control group without fractures)**			
	Patients (*n* = 155)	Men (*n* = 22)	Women (*n* = 133)
Age	Ø 70.62	Ø 71.09	Ø 70.54
(in years)	(min.: 61; max.: 85)	(min.: 61; max.: 78)	(min.: 61; max.: 85)
BMI	Ø 25.3	Ø 27.4	Ø 24.95
(kg/m^2^)	(min.: 17.6; max.: 37.6)	(min.: 21.6; max.: 37.6)	(min.: 17.6; max.: 32.1)

## Data Availability

The data presented in this study are available on request from the corresponding author due to restrictions imposed by the institutional ethics committee and local data protection regulations, which do not allow open sharing of individual-level clinical and imaging data.

## Abbreviations

The following abbreviations are used in this manuscript:

AUC	Area under the curve
BMD	Bone mineral density
BMI	Body mass index
CTXA	Computed tomography X-ray absorptiometry
DEXA	Dual-energy X-ray absorptiometry
FFP	Fragility fractures of the pelvis
FFS	Fragility fractures of the sacrum
GE	General Electric
HU	Hounsfield unit
LS	Lumbar spine
LV	Lumbar vertebrae
MA	Mean age
MRI	Magnetic resonance imaging
*n*	Number
OVF	Osteoporotic vertebral fracture
prox.	proximal
Q1-Q3	First and third quartile
QCT	Quantitative computed tomography
ROC	Receiver operating characteristic
ROI	Region of interest
SD	Standard deviation
SIF	Sacral insufficiency fracture
STIR	Short tau inversion recovery
TEP	Total endoprosthesis
TH	Thoracic vertebrae
